# Evaluation of binding property of mucilage from *Litsea glutinosa* wall

**DOI:** 10.4103/0974-8490.72325

**Published:** 2010

**Authors:** Sunil K. Mishra, A. Kumar, A. Talukdar

**Affiliations:** *Department of Pharmaceutics, Institute of Technology, Banaras Hindu University, Varanasi - 221 005, India*; 1 *Department of Herbal Technology, Itanagar, Arunachal Pradesh, India*

**Keywords:** *Litsea glutinosa*, mucilage, polysaccharide

## Abstract

**Background:**

Litsea glitinosa is an evergreen tree of medium size which grows to a height of about 20 to 30 feet. It belongs to family Lauraceae. In India it is found mainly in North Eastern region. The leaves and the mucilage from the bark of plant is utilized in the gum for poultices.

**Methods:**

Mucilage of Litsea glutinosa was isolated from powdered bark by continuous hot extraction technique using water and precipitation by absolute alcohol (38% w/w yield). The mucilage was evaluated for binding properties in tablets and granules, using paracetamol as a model drug. The granules were prepared using 4 different concentrations of mucilage (4%, 6%, 8%, and 10%) and evaluated for percentage of fines, average particle size, total porosity, compressibility index, and flow properties. The prepared tablets were evaluated for content uniformity, hardness, friability, disintegration time, and *in vitro* dissolution profiles.

**Results:**

The results obtained with the lower concentrations of mucilage, that is, less than 6% were not so encouraging. The tablets prepared by using 10% mucilage of *L. glutinosa* as binder exhibited more hardness as compared with the starch.

**Conclusion:**

It may be concluded that the concentration ranging from 6% to 8% of *L. glutinosa* mucilage may be considered as better option as a binding agent for the preparation of tablets as compared to the starch (10%).

## INTRODUCTION

*Litsea glutinosa* is an evergreen tree of medium size, which grows to a height of about 20-30 ft. It belongs to the family Lauraceae. *L. glutinosa*is found in mixed primary and secondary forest and thickets. Distributed from India through Indo-China toward the Malesian area where it grows in all parts, and northern Australia. In India it is found mainly in North Eastern region. The seeds contain an aromatic oil, which has been used to make candles and soap. The roots yield fibers used in Thailand for rope manufacture and for paper pulp. The fruits have a sweet creamy edible pulp that can be taken as food. The young leaves are used as fodders. The powdered seeds are also applied medicinally against boils. The leaves and the mucilage in the gum from the bark have been used for poultices. The bark also acts as a demulcent and mild astringent in diarrhea and dysentery.[1] The essential oil of the plant is reported to have psychopharmacologic actions.[2] Kar *et al* also reported that the essential oils of *L. glutinosa* have beneficial effects on the isolated tissue of the cardiovascular system.[3] The methanolic extract of the bark have antibacterial activity against 16 different microorganisms as reported by Mandal *et al*.[4] The bark of the plant *L. glutinosa* also contains alkaloids that have been identified and reported by Yang *et al*.[5]

Mucilages of *L. glutinosa* is a hetero-polysaccharide, polyuronides consisting of sugar and uronic acid units.[6] They are usually formed from the cell wall or deposited on it in layers. They swell in water and form a gel.[6] The usefulness of mucilages as emulsifying, gelling, and suspending agents has been well documented.[7] Some of the mucilages have also been used in tablet formulations as binding agents and also to sustain the drug release.[8] The present investigation is carried out to study the binding property of the mucilage obtained from *L. glutinosa* in tablet manufacturing.

## MATERIAL AND METHODS

### Plant material

The bark of *L. glutinosa* was collected from Guwahati. The barks were cut into small pieces and dried in a hot air oven at 60°C. The chips were powdered in a mechanical grinder. Paracetamol was used as a model drug in the study. All the chemicals and other reagents used in the study were of AR grade.

### Isolation of mucilage

The weighed quantity (30 g) of bark powder was extracted with solvent ether in a soxhalate apparatus for about 6–8 h. The marc was dried and macerated overnight with absolute alcohol. The supernatant liquid was discarded after maceration. Again the defatted and decolorized powder was macerated for about 18–20 h with 5% aqueous acetic acid solution. Maceration process was repeated three times for complete extraction of mucilage. The extract was filtered and concentrated to about 50 mL by evaporation on a water bath. Lastly, the mucilage was precipitated completely by the addition of an excess of absolute alcohol. The mixture was filtered and washed repeatedly with absolute alcohol. The filtrate was dried till constant weight at 100œC and the percentage of mucilage with reference to air-dried bark was calculated. The percentage of mucilage found was 38% w/w. The mucilage was powdered and passed through sieve number 80. The powdered mucilage was stored in a desiccator until further use.[9]

### Physicochemical and microbial properties of mucilage

The physicochemical properties, such as solubility, swelling index, loss on drying, viscosity, and microbial load of the mucilage, were determined according to the Indian pharmacopoeial procedures.[10] The pH of the mucilage was determined using a digital pH meter.

### Preparation and evaluation of granules

Paracetamol was used as a model drug to formulate granules. Starch was used as disintegrant, whereas lactose and talc were used as diluent and lubricant, respectively, as per the guidelines of standard text. The binder solution was prepared by dissolving the mucilage of *L. glutinosa* in water at 4%, 6%, 8%, and 10% w/v concentrations. The granules of batch size 150 g were prepared by wet granulation method.[11,12] The drug, lactose, talc, and starch were mixed thoroughly, and a sufficient volume of ~ 30 mL of 4%, 6%, 8%, and 10% w/v of mucilage of *L. glutinosa* was added slowly to the powder blend, and kneading was performed for ~ 10 min until formation of a dough mass with enough cohesiveness. The dough mass was forced through a sieve no. 16 (1180 µm) and dried at 50°C in a hot air oven for 12 h. The dried granules were re-sieved through a sieve no. 20 (850 µm). The prepared granules were then evaluated for percentage of fines, particle size, and flow properties (by measurement of angle of repose).[13,14] The bulk and tapped densities of the granules were assessed in accordance with the *USP* 25 using a tapped volumeter apparatus. Compressibility index of the granules was determined by Carr’s compressibility index.[13–15] Total porosity was determined as described by measuring the volume occupied by selected weight of a powder and the true volume of granules.[13–15]

### Preparation and evaluation of tablets

The tablets were compressed by using single punch machine with concave-faced punches. A batch size of 100 tablets was prepared. The prepared tablets were evaluated for content uniformity, hardness, friability, disintegration time, and *in vitro* dissolution profile by Indian Pharmacopoeia 1996 method.[16]

## RESULTS AND DISCUSSION

The dried and coarsely powdered tubers of *L. glutinosa* yielded a high percentage (38% w/w) of mucilage using absolute alcohol as mucilage–precipitating solvent. The physicochemical properties of mucilage were determined and shown in Table 1.The extracted and purified mucilage was evaluated for pH, which was found to be 7.2.

**Table 1:** Physicochemical Property of *L. glutinosa* (Bark)

Parameter (S)	Result (S)
Solubility	Swells in cold water
	considerably but Quickly
	dissolves in warm water forming
	Viscous colloidal solution.
	Insoluble in Ethanol, chloroform
	and ethyl acetate.
Swelling index	14%
P^H^	7.25
Loss on drying	6.8%
Microbial load	
a. Bacteria	94
(no. of CFU/gm. Mucilage)	
b. Fungi	
(no. of CFU/gm. Mucilage)	110

The prepared granules were evaluated for percentage of fines, particle size, and flow properties [Table 2]. It was observed that the percentage of fines was reduced as the concentration of mucilage was increased. The percentage of fines was a little higher in granules prepared using 6% w/v mucilage of *L. glutinosa* as binder but 8% concentration may be considered good as compared with the starch of 10% w/v. The flow property of granules was determined by *angle* of repose, which was found to be 30° to 32°. The mean particle size (between 0.31 and 0.38 mm) was found to be satisfactory for preparation of tablets. Hence all the granules exhibited good flow properties [Table 2]. The bulk densities of the prepared granules were found to decrease significantly by increasing the concentrations of *L. glutinosa* mucilage and was found to be lowest (0.498 ± 0.037) at 10% w/v. This result may be due to the formation of larger agglomerates and the decrease in fines in the granules, because increasing the concentrations of mucilage provides more binding to the granules. The compressibility index [Table 2] indicates a decrease in flowability with increasing *L. glutinosa* mucilage. In general, compressibility index values up to 15% result in good to excellent flow properties.[17] Percentage porosity values of the granules ranged from 30.29% to 38.32%, indicating that the granules are loosely packed and confirming that the particles are not of variable sizes. In general, a percentage porosity value below 26% shows that the particles in the powders are of variable sizes, and a value greater than 48% shows that the particles in the powder are in the form of aggregates or flocculates.[18] All the results indicate that the granules prepared using different concentrations (4%, 6%, 8%, and 10%) possess satisfactory flow properties, compressibility, and porosity.

**Table 2:** Properties of granules from different conc. of *L. glutinosa* mucilage & starch

Property (S)	*Litsea glutinosa* mucilage as binder				Starch
	4%	6%	8%	10%	10%
Percentage of fines	25.00	19.22	16.60	14.50	17.88
Mean Particle size (mm)	0.33	0.39	0.42	0.48	0.42
Angle of repose (°)	28°	30°	30°	30°	32°
Loose bulk density (g/cm^2^) ± SD	0.624 ± 0.045	0.540 ± 0.028	0.511 ± 0.03	0.050 ± 0.042	0.489 ± 04
Tapped bulk density (g/cm^2^) ± SD	0.522 ± 0.06	0.515 ± 0.032	0.508 ± 0.052	0.498 ± 0.037	0.562 ± 0.332
Compressibility Index(%) ± SD	8.11 ± 0.92	8.92 ± 0.22	11.56 ± 0.67	13.52 ± 0.123	13.42 ± 1.01
Total porosity (%) ± SD	25.23 ± 2.23	32.02 ± 2.32	38.83 ± 3.32	38.87 ± 3.37	36.46 ± 2.63

Four batches of 100 tablets were prepared with mucilage of *L. glutinosa* of different concentrations (4%, 6%, 8%, and 10%) and evaluated for content uniformity, hardness friability, disintegration time, and *in vitro* dissolution profiles, and so on [Table 3].

**Table 3:** Properties of tablets from different conc. of *L. glutinosa* mucilage & starch

Property (S)	*Litsea glutinosa* mucilage as binder				Starch
	4%	6%	8%	10%	10%
Content uniformity (%) ± SEM	88.68 ± 0.33	96.82 ± 0.34	97.22 ± 0.42	98.68 ± 0.32	98.01 ± 0.42
Hardmess (Kg/cm^2^) ± SEM	4.82 ± 0.02	6.10 ± 0.10	6.80 ± 0.08	8.22 ± 0.08	6.50 ± 0.08
Percentage friability	0.5	0.3	0.2	0.14	0.22
Disintegration time (Sec)	220	280	322	350	248

The tablets exhibited good content uniformity. The hardness of tablets increased with increase in the percentage of binding agent. The tablets prepared with 10% mucilage of *L. glutinosa* were showing more hardness compared with tablets prepared with 10% starch mucilage. The percentage friability values were constant in all the batches of tablets prepared by using different concentrations of mucilage. Disintegration time of 10% w/v concentration of *L. glutinosa* mucilage was higher in comparison with the tablets prepared by using 10% w/v of starch mucilage.

**Figure 1:**
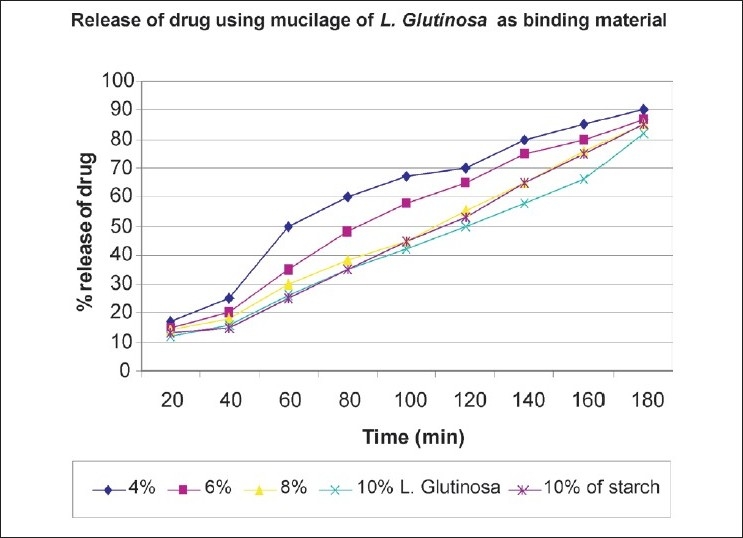
Comparison of release of paracetamol prepared with *L. glutinosa* and Starch in 0.1 M HCl. Each data represents the mean ± S.E of five experiments

The dissolution profile (*in vitro*) indicates that the 8% w/v concentration of *L. glutinosa* mucilage is more uniform and almost more than 80% of the drug gets released within 3 h, but in the 10% concentration, drug release is slightly less [Figure 1]. The trend of drug release was found decreasing with increase in concentration of *L. glutinosa* mucilage. The result showed that the drug release from the tablets prepared using mucilage of 4%, 6%, and 8% w/v concentrations of *L. glutinosa* mucilage was more than 85% in 180 min [Figure 1].

It has been observed from the study that the bark of the plant *L. glutinosa* has high mucilage content (38% w/w of dried bark),so the plant may be taken as a good natural source of mucilage. The results also show that the mucilage of *L. glutinosa* has better binding property as compared with starch and it is also economical compared with starch. Furthermore, *L. glutinosa* mucilage can also be evaluated for sustained drug release from tablets, since the tablets prepared using mucilage of *L. glutinosa* produced a sticky film of hydration on the surface, which may reduce the drug release rate. Hence *L. glutinosa* mucilage can be evaluated for its efficacy in the formulation of sustained release tablets.
